# Highly Concentrated Alginate-Gellan Gum Composites for 3D Plotting of Complex Tissue Engineering Scaffolds

**DOI:** 10.3390/polym8050170

**Published:** 2016-04-26

**Authors:** Ashwini Rahul Akkineni, Tilman Ahlfeld, Alexander Funk, Anja Waske, Anja Lode, Michael Gelinsky

**Affiliations:** 1 Centre for Translational Bone, Joint and Soft Tissue Research, University Hospital Carl Gustav Carus and Faculty of Medicine of Technische Universität Dresden, 01307 Dresden, Germany; ashwini_rahul.akkineni@tu-dresden.de (A.R.A.); tilman.ahlfeld@tu-dresden.de (T.A.); anja.lode@tu-dresden.de-dresden.de (A.L.); 2 IFW Dresden, Institute for Complex Materials, P.O. 270116, 01171 Dresden, Germany; a.funk@ifw-dresden.de (A.F.); a.waske@ifw-dresden.de (A.W.)

**Keywords:** 3D plotting, 3D printing, rapid prototyping, additive manufacturing, biofabrication, alginate, gellan gum, hydrogels, biopolymers, composites

## Abstract

In tissue engineering, additive manufacturing (AM) technologies have brought considerable progress as they allow the fabrication of three-dimensional (3D) structures with defined architecture. 3D plotting is a versatile, extrusion-based AM technology suitable for processing a wide range of biomaterials including hydrogels. In this study, composites of highly concentrated alginate and gellan gum were prepared in order to combine the excellent printing properties of alginate with the favorable gelling characteristics of gellan gum. Mixtures of 16.7 wt % alginate and 2 or 3 wt % gellan gum were found applicable for 3D plotting. Characterization of the resulting composite scaffolds revealed an increased stiffness in the wet state (15%–20% higher Young’s modulus) and significantly lower volume swelling in cell culture medium compared to pure alginate scaffolds (~10% *vs.* ~23%). Cytocompatibility experiments with human mesenchymal stem cells (hMSC) revealed that cell attachment was improved—the seeding efficiency was ~2.5–3.5 times higher on the composites than on pure alginate. Additionally, the composites were shown to support hMSC proliferation and early osteogenic differentiation. In conclusion, print fidelity of highly concentrated alginate-gellan gum composites was comparable to those of pure alginate; after plotting and crosslinking, the scaffolds possessed improved qualities regarding shape fidelity, mechanical strength, and initial cell attachment making them attractive for tissue engineering applications.

## 1. Introduction

Since the discovery of gellan gum, an anionic polysaccharide produced by the bacteria *Sphingomonas paucimobilis*, it has found wide spread applications in food industry as a gelling/stabilizing agent and also in plant and bacterial culture systems [1,2,3,4]. In the recent decades, its suitability for tissue engineering approaches has been studied because of its versatile gelling properties, *i.e.*, thermo-reversible gelling at near body temperature and ionotropic gelling by low concentrations of mono-, di- or trivalent cations. Gellan gum readily dissolves in water or aqueous solutions at higher temperatures (more than ~40 °C) at which the polymer chains exist as disordered coils. When cooling down, they form ordered helical structures resulting in a weak thermo-reversible gel. A stiffer, thermally irreversible gel is formed when cations (e.g., Ca^2+^) are added [3,5,6]. Then, cation bridges are formed between the carboxyl groups of the d-glucuronosyl residues by ionic bonding, strongly increasing the gel strength [5].

Alginate, a polysaccharide isolated from brown algae, has been extensively used for tissue engineering applications for a long time. Alginate does not form a thermo-responsive gel, but can be crosslinked with multivalent cations. At low concentrations, both alginate and gellan gum have been often used for embedding drugs, growth factors and cells; such loaded gels can be injected through minimally invasive methods to regenerate defect sites [7,8,9]. However, at low concentrations and by simple injecting methods, defects of critical size could not be successfully repaired due to limited diffusion of nutrients/wastes within and limited tissue ingrowth into the hydrogel matrix as well as low mechanical strength in the long term. Fabricating a shaped 3D structure with defined porosity, yet mechanically robust over long time periods *in vivo* would enable the challenges involved in repair of large defects to be overcome. In addition, shaping hydrogels into channel- or tube-like structures would significantly bring forward tissue regeneration approaches, e.g., with respect to vascularization or vascular tissue engineering.

In the recent decades, additive manufacturing (AM) technologies have made considerable progress in addressing challenges in tissue engineering [10,11]. Precise positioning of biomaterials (without or loaded with biological components) has opened up the possibility of fabricating constructs for critical sized defects having the desired architecture. Furthermore, the integration of medical imaging with AM technologies has made it possible to fabricate patient specific scaffolds [12]. Based on three general principles, various AM techniques have been evolved and found their application in hydrogel-based tissue engineering: laser-based, extrusion-based, and inkjet-based systems [11]. 3D plotting is an extrusion-based technique in which pasty or highly viscous materials are used to fabricate a porous 3D construct in a computer aided design/manufacturing (CAD/CAM) process [13].

For processing by extrusion techniques like 3D plotting, the starting materials must have certain intrinsic properties such as high print fidelity and suitable stabilization mechanisms to allow fabrication of 3D structures with the desired internal and external architecture. Good print fidelity is achieved with high viscosity, shear thinning behavior, and high yield stress. Also, the stabilization procedure after printing plays an important role in maintaining print fidelity [14]. Low concentrated alginate or gellan gum solutions have been used by 3D extrusion methods for scaffold based tissue engineering. However, their strong limitation is the lack of sufficient print fidelity owing to low viscosity. Hence, often chemical modifications are required to tune the rheological properties or for stabilization procedures during/after printing [4,15,16,17].

An alternative for fabrication of designed hydrogel scaffolds is the usage of high polymer concentrations to attain sufficient high viscosity for printing [18,19,20]. We have recently demonstrated that highly concentrated pastes (16.7% alginate and alginate/gelatin blends) can be processed by 3D plotting to fabricate scaffolds with high shape fidelity having clinically relevant sizes [19,20]. Also hollow tubes, which can be assembled to 3D scaffolds, were successfully fabricated by using highly concentrated alginate pastes [19]. Whereas drugs and growth factors can still be easily mixed into the pastes prior to plotting, the integration of cells into the stiff material is not possible and hence, cell colonization of such scaffolds depends on cell adhesion on the strand surface [18,20].

However, as ionic crosslinking of alginate is reversible, exchange of Ca^2+^ ions from the alginate matrix to the cell culture medium at physiological conditions [21] would weaken the scaffolds made of highly concentrated alginate affecting their mechanical stability as well as their swelling degree. Hence, alginate gels require constant high concentrations of cations to maintain structural stability for long-term applications. In contrast, Smith *et al.* showed that for crosslinking of gellan gum, cation concentrations only in the range of ~5 mM (similar to cation concentrations in cell culture medium) are required [3]. Gellan gum can be crosslinked by simply adding the cell culture medium to the gels and the ion-crosslinked gels are mechanically stable for long terms in *in vitro* conditions.

Based on the hypothesis that mixing of alginate with gellan gum would result in a hydrogel composite whose stability is influenced by both alginate and gellan gum crosslinking, the intention of the present study was to combine the intrinsic advantages of both the materials—*i.e.*, excellent processability of 16.7% alginate and enhanced stability of gellan gum gels. High concentrated composite pastes were developed and evaluated for their viscosity and processability by 3D plotting. In order to investigate the impact of the added gellan gum on scaffold properties, pure alginate and composite scaffolds were fabricated and characterized for their porosity, swelling behavior and mechanical properties. Finally, the response of human mesenchymal stem cells when cultured for 14 days on the scaffolds was evaluated.

## 2. Experimental Section

### 2.1. Preparation of Plotting Pastes and Viscosity Measurements

Prior to composite preparation, alginate (Sigma-Aldrich, Steinheim, Germany) and gellan gum (Gelzan; Sigma-Aldrich, Steinheim, Germany) powders were sterilized by super critical carbon dioxide sterilization method as described [22]. An amount of 2 and 3 wt % gellan gum was dissolved in deionized water by continuous stirring at 80 °C for 1 h. For preparation of the composite materials, alginate power was homogeneously mixed using both 2 and 3 wt % gellan gum solution (AGG2 and AGG3) to attain a final concentration of 16.7 wt % alginate. A pure 16.7 wt % alginate paste (PA) was prepared by using phosphate buffered saline (PBS; Gibco, Life technologies, Bleiswijk, Netherlands) instead of a gellan gum solution. For 3D plotting, the pastes were then loaded into cartridges (Nordson EFD, Bedfordshire, UK) and centrifuged at 300× *g* for 15 min to remove trapped air bubbles.

Viscosity of the individual pastes was measured using a rotary rheometer (Rheotest^®^ RN 4.1, Medingen, Germany) with a cone/plate having an angle of 1°. A constant shear rate of 10 s^−1^ was applied for 300 s and the corresponding viscosity was obtained for the individual pastes. Shear thinning experiments were done by applying a shear rate of 0–100 s^−1^ (with an increment of 0.08 s^−1^) over a period of 1200 s and the corresponding viscosity was obtained.

### 2.2. Scaffold Fabrication

Scaffolds were fabricated using a 3D plotting device (BioScaffolder 2.1, GeSiM mbH, Grosserkmannsdorf, Germany) operated under sterile conditions in a laminar flow bench. Briefly, the paste was extruded, by applying air pressure, from the cartridge through a dosing needle (Globaco, Roedermark, Germany) having an inner diameter of 840 µm. While extruding the paste, the print head moved according to a predefined CAD data set to deposit the paste layer by layer to build the 3D scaffold. The plotted scaffolds were then transferred into a 1 M CaCl_2_ solution for crosslinking of alginate and gellan gum. The stabilized scaffolds were either frozen at −20 °C for 12 h and thereafter freeze dried (Alpha 1-2, Christ, Osterode am Harz, Germany) for 8 h or immediately used for the experiments.

### 2.3. Scaffold Characterization

Freeze dried scaffolds with a size of 6.5 × 6.5 × 7 mm^3^ (composed of 25 layers with 5 strands per layer having a 0°/90° lay down pattern) were used for measurement of the total scaffold porosity with a helium gas pycnometer (Ultrapyc 1200e, Quantachrome, Boynton Beach, FL, USA). The quotient of apparent (ρ_app_) and pycnometrically obtained density (ρ_pyc_) was used to calculate the total porosity using the formula:

$$\text{Porosity }\left[\text{vol}-\%\right]=\left(1-\frac{\rho_{\text{app}}}{\rho_{\text{pyc}}}\right)\times 100\%$$

Micro-computed tomography (µ-CT) imaging was done on scaffolds (having a size of 6.5 × 6.5 × 2.7 mm^3^, composed of 8 layers with 5 strands per layer having a 0°/90° lay down pattern) using vivaCT 75 (Scanco Medical, Brüttisellen, Switzerland) with X-ray energy of 45 keV, intensity of 177 µA and voxel resolution of 10.5 µm. 200 image slices (corresponding to ~2 mm of the scaffold height) obtained from the µ-CT were used to calculate the porosity of the scaffolds using ImageJ 1.44p (National Institutes of Health, Bethesda, MD, USA).

Single freeze-dried strands were non-destructively tested by high resolution X-ray computed tomography (Nano-CT) using a Phoenix Nanotom m device by General Electric (GE, Wunstorf, Germany). Three strands of each type (PA, AGG2 and AGG3) were scanned over a strand length of about 5 mm. Scanning parameters were kept identical for each sample at an acceleration voltage of U = 70 kV, current of *I* = 160 µA, exposure time of *t* = 500 ms. A number of 1400 images were acquired for each scan over an angle range of 360° and at a magnification of M = 50. 3D volume datasets were reconstructed from the acquired 2D X-ray projections with GE software “datos 2.2” at a voxel size of about *v*_x_ = 2 µm. The 3D volume analysis software “Avizo 9” (FEI Corporate headquartes, Hillsboro, OR, USA) was used for X-ray scan visualization and porosity measurements. The analyzed region of interest of the scans was 4 mm long. An unsharp masking and median filter were used for feature sharpening and noise reduction. For porosity measurements of single scans, the threshold defining dense material was found by using the ISO50 value of the dataset’s histogram peaks as initial threshold but adjusted manually for optimal results. The “fill holes” algorithm implemented in Avizo delivered the volume of the porous space, which together with the volume of the dense material led to the calculated internal porosity of the strands.

Freshly crosslinked (FC) scaffolds (having a size of 6.5 × 6.5 × 7 mm^3^, composed of 25 layers with 5 strands per layer having a 0°/90° lay down pattern) were imaged by light microscopy using a stereo microscope (Leica M205C, Leica Microsystems, Wetzlar, Germany) before and after wetting in cell culture medium (composition see Section 2.4.1.), Hank’s balanced salt solution (HBSS, with total Ca and Mg content of 1.26 and 0.89 mM respectively; Gibco, Life technologies, Bleiswijk, Netherlands) or PBS for 24 h. The dimensions of the strands in the dry and wet state were measured from the corresponding images using ImageJ 1.44p (NIH, Bethesda, MD, USA) to calculate the swelling ratio. For scanning electron microscopy (SEM) analysis, the freeze dried samples were coated with gold (Leica EM SCD005, Leica Microsystems, Wetzlar, Germany) and imaged using a Philips XL 30/ESEM with field emission gun (FEG), operated in SEM mode. Compressive strength of the scaffolds was measured using a universal testing machine (Z010 equipped with a 10 kN and 100 N load cell, Zwick, Ulm, Germany). Scaffolds in the dry state were initially subjected to a preload of 2 N and then a static compressive load was applied in the z direction at a rate of 1 mm·min^−1^. For wet state testing, the scaffolds incubated in HBSS with Ca^2+^ and Mg^2+^ for 24 h at 37 °C were subjected to compressive loading. In the case of wet (re-swollen) state testing, a static compressive load of 1 mm·min^−1^ with a preload of 0.1 N was applied on the scaffolds having a size of *ca.* 6.9 × 6.9 × 7.3 mm^3^. The corresponding compressive modulus was calculated from the data obtained.

### 2.4. Cultivation of hMSC on the Plotted Scaffolds

#### 2.4.1. Cell Seeding and Osteogenic Differentiation

Human mesenchymal stem cells (hMSC), isolated from bone marrow aspirates of healthy donors after obtaining informed consent, were kindly provided by Medical Clinic I of Dresden University Hospital *Carl Gustav Carus* (Prof. Bornhäuser and co-workers). The application of hMSC for *in vitro* experiments was approved by the ethics commission of Technische Universität Dresden (EK263122004, date of approval: 10-12-2004). The hMSC were expanded and cultured in α-MEM containing 15% fetal calf serum (FCS), 1% l-glutamate, 100 U/mL penicillin, and 100 mg/mL streptomycin (all from Biochrom, Berlin, Germany) at 37 °C and 5% CO_2_. The scaffolds for cell culture were fabricated with a mismatch lay down pattern *i.e.*, every alternate layer was plotted with an offset of 840 µm relative to the underneath parallel layer such that the pores of the scaffold in perpendicular direction were not straight but angled in order to increase the cell seeding efficiency . The scaffolds with a size of *ca.* 6 × 6 × 3.2 mm^3^ (4 layers with 4 strands per layer) were incubated in cell culture medium for 48 h prior to seeding without freeze drying and thereafter seeded with hMSC (passage 4) at a density of 0.5 × 10^5^ cells per scaffold. Osteogenic differentiation of the hMSC was induced 1 day after cell seeding by adding osteogenic supplements (10^−7^ M dexamethasone, 10 mM β-glycerophosphate, 0.05 mM ascorbic acid 2-phosphate, and 10 nM Vitamin D3; all from Sigma-Aldrich, Steinheim, Germany) to the medium.

#### 2.4.2. Biochemical Analysis of Cell Proliferation and Osteogenic Differentiation

After 1, 7, and 14 days of culture, the number of cells attached and grown on the scaffolds was evaluated by DNA quantification; osteogenic differentiation was analyzed by measurement of alkaline phosphatase (ALP) activity. As previously described [23], the samples were washed with PBS and frozen at −80 °C; for cell lysis, the thawed samples were incubated in 1% Triton X-100 (Merck, Darmstadt, Germany) in PBS and treated by ultrasonication. The DNA content of the cell lysates was determined with the QuantiFluor dsDNA system (Promega, Madison, WI, USA) according to the manufacturer’s instructions and correlated with the cell number using a calibration line. For measurement of ALP activity, another aliquot of each cell lysate was mixed with 1 mg·mL^−1^ *p*-nitrophenylphosphate (Sigma-Aldrich, Steinheim, Germany) in 0.1 M diethanolamine/1% Triton X-100/1 mM MgCl_2_ (pH 9.8). After incubation at 37 °C for 30 min, the enzymatic reaction was stopped by addition of 1 M NaOH; *p*-nitrophenolate (pNp) formation was quantified by measuring the absorbance at 405 nm. The amount of pNp was calculated using a *p*-nitrophenol calibration line and related to the cell number in each sample (calculated from the DNA content) to yield specific ALP activity (µmol pNp/30 min/10^6^ cells).

#### 2.4.3. Confocal Laser Scanning Microscopy (cLSM)

After 1, 7, and 14 days of culture, samples were fixed with 3.7% formaldehyde in PBS for 1 h, followed by staining the nucleus and cytoskeleton of the cells with Hoechst 33342 (Invitrogen, OR, USA) and phalloidin-Alexa Fluor 488^®^ (Invitrogen, Carlsbad, CA, USA), respectively. The stained samples were imaged using a Leica TCS SP5 confocal microscope (Leica Microsystems, Wetzlar, Germany) located in the *MTZ Imaging Facility* of Technische Universität Dresden (Dresden, Germany) to assess the cell distribution and morphology on the scaffolds.

### 2.5. Statistics

All experiments were performed using replicates (*n* = 3–20, indicated in the figure captions). The results were expressed as mean ± standard deviation (SD). One-way ANOVA was performed to test for statistical significance, *p* < 0.05 was considered as significantly different.

## 3. Results

### 3.1. Properties of the Composite Pastes

In contrast to the pure alginate (PA) paste, the composite pastes of alginate and gellan gum (AGG2, AGG3) started to gel a few minutes after the preparation was completed and the pastes cooled down to room temperature. However, the hardening of the composites did not impair the 3D plotting process.

**Figure 1:**
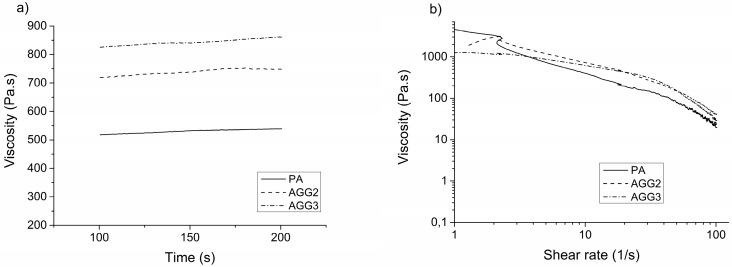
Representative images of single measurements of (**a**) viscosity of the pastes measured at a constant shear rate of 10·s^−1^; (**b**) viscosity measured as function of shear rate (0–100 s^−1^).

Viscosity as an important parameter for 3D extrusion was studied with a rotary viscosimeter by applying a constant as well as an increasing shear rate on the pastes (Figure 1). The viscosity of the alginate-gellan gum composite pastes was found to be higher than that of the pure alginate paste. Correspondingly, the air pressure required to extrude the composite pastes was higher than that of pure alginate (summarized in Table 1). Increase of the shear rate revealed a shear thinning behavior for all the pastes.

### 3.2. Characterization of the Scaffolds

**Figure 2:**
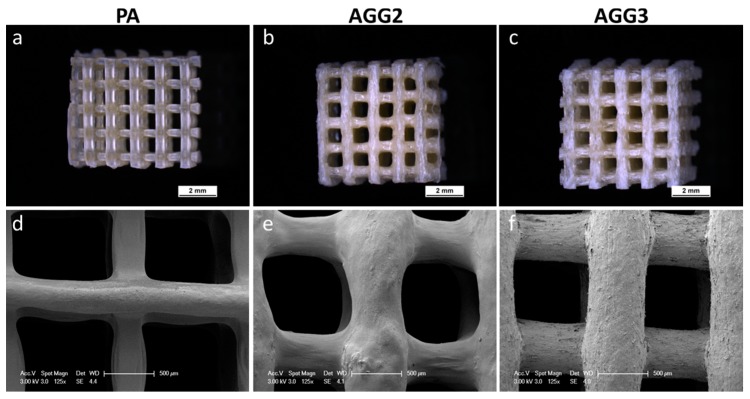
Light microscopic images of plotted scaffolds (25 layers) after freeze drying (**a**–**c**) (scale bar = 2 mm) and scanning electron microscopy (SEM) images of the scaffold surface (**d**–**f**; scale bar = 500 µm).

**Figure 3:**
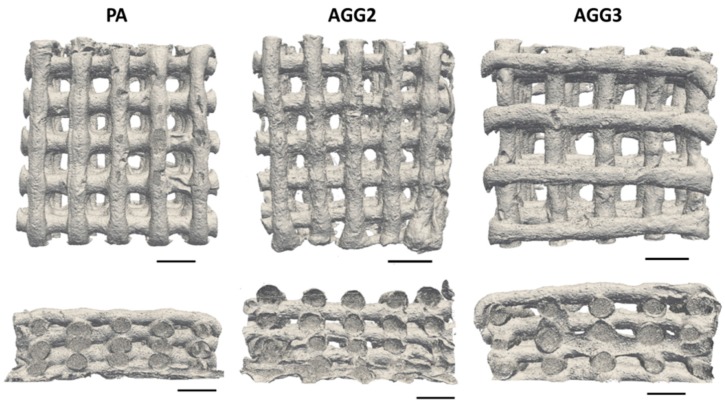
Reconstructed micro-computed tomography (µ-CT) images of the plotted scaffolds in freeze-dried state (top and side views; scale bar = 1 cm).

Immediately after crosslinking the plotted scaffolds, no optical distinctions between the different scaffold types were noticeable. However, after freeze drying, the strands of the pure alginate scaffolds had a brownish glassy appearance whereas the strands of the alginate-gellan gum composite scaffolds had a shiny whitish appearance (Figure 2a–c). Microscopic analyses revealed that PA scaffolds showed higher shrinkage upon freeze drying compared to composite scaffolds: the thickness of the PA strands is clearly reduced in comparison to the composite strands. Interestingly, the cylindrical strand shape for AGG3 appeared to be better preserved after freeze drying in comparison to PA and AGG2 scaffolds (Figure 2d–f). Micro-CT images show similar observations *i.e.*, higher shrinkage of PA scaffolds compared to AGG scaffolds. Also, µCT images (Figure 3) show many collapsed lateral pores in the PA scaffolds in contrast to AGG scaffolds indicating that the addition of gellan gum increased the shape fidelity.

Measurement of the total open porosity of freeze-dried scaffolds revealed that PA scaffolds had a significantly higher porosity when compared to AGG2 and AGG3 scaffolds (Table 2). Porosity calculated from µ-CT data demonstrated the same trend concerning the different types of scaffolds but lower values. Due to the limited resolution of the µ-CT measurements the internal porosity of the dry biopolymer strands cannot be assessed and therefore did not contribute to the total porosity values, in contrast to the results achieved by pycnometric measurements.

**Figure 4:**
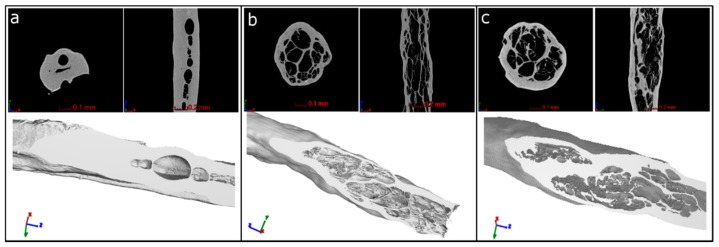
NanoCT images of a cross section and logitudinal section of a single strand of (**a**) PA; (**b**) AGG2; (**c**) AGG3. Bottom images: 3D reconstructed images of the CT scans with applied slice filter.

From Nano-CT scans of the single strands, it was possible to get a visual impression of the strand’s inner structure and porosity. PA samples had a low internal porosity in the range of 4%–12% with bubble like pores (Figure 4a) in a size range of about d = 30 to 300 µm (sphere equivalent diameter) (Supplementary Materials, Figure S1a). AGG2 and AGG3 showed significantly higher internal porosities between 32% and 42% compared to PA. Also, the pore shape was fundamentally different to that of PA samples. The pores found in AGG2 and AGG3 are approximately spherical, but each of them is subdivided into smaller cells by thin material walls forming a highly connected pore network with a foam-like structure (Figure 4b,c). These pore cells are in the size range of about *d* = 20–200 µm in sphere equivalent diameter (Supplementary Materials Figure S1b,c).

**Figure 5:**
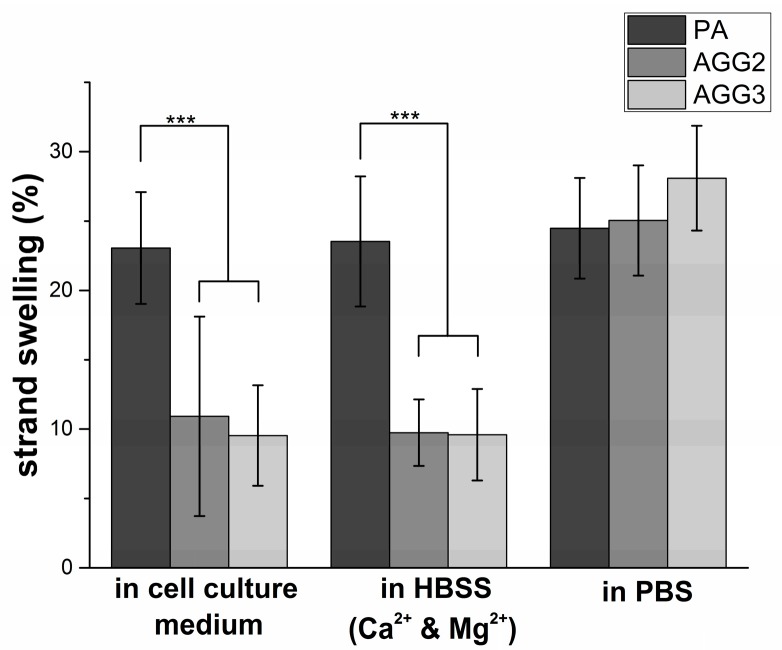
Strand swelling calculated by measuring the strand diameters before and after incubation of freshly crosslinked scaffolds in the respective solutions. (mean ± SD; *n* = 20; *** *p* < 0.001).

Strand diameter increase by swelling of the scaffolds was calculated after incubating them in different solutions for 24 h at 37 °C (Figure 5). Pure alginate strands showed a constant swelling ratio when incubated in all the solutions (23.1% ± 4.0%; 23.5% ± 4.7%, and 24.5% ± 3.6% in cell culture medium, HBSS with Ca^2+^ and Mg^2+^ and PBS, respectively). In contrast, swelling of strands of the alginate-gellan gum composite scaffolds was lower when incubated in cell culture medium (AGG2 = 10.92% ± 7.19%; AGG3 = 9.74% ± 2.39%) and HBSS with Ca^2+^ and Mg^2+^ (AGG2 = 9.74% ± 2.39%; AGG3 = 9.58% ± 3.29%). However, the composite scaffolds showed a 2-fold increase in strand swelling when incubated in PBS (AGG2 = 25.04% ± 3.97%; AGG3 = 28.09% ± 3.77%). In fact, the strand swelling in PBS was even higher in comparison to that of pure alginate scaffolds.

**Figure 6:**
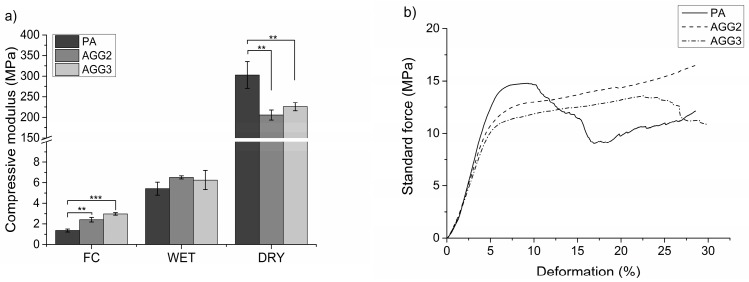
Young’s moduli of (**a**) freshly crosslinked (FC) scaffolds, scaffolds in re-swollen state (WET) after freeze drying and in dry state (DRY) after freeze drying (mean ± SD; *n* = 5; ** *p* < 0.005, *** *p* <0.001); (**b**) Representative stress strain curves of PA and composite scaffolds in dry state.

Young’s modulus was calculated from the data obtained from compressive tests performed on the scaffolds immediately after crosslinking and after freeze drying in both dry and wet (re-swollen) state (Figure 6a). After crosslinking, the composite scaffolds showed higher moduli (AGG2 and AGG3 = 2.14 ± 0.23 MPa and 2.97 ± 0.12 MPa, respectively) compared to PA scaffolds (1.36 ± 0.14 MPa). However, in dry state (after freeze drying) the PA scaffolds had with 302.8 ± 32.6 MPa a higher Young’s modulus in comparison to AGG2 (205.7 ± 12.0 MPa) and AGG3 (225.9 ± 9.9 MPa). Interestingly, in wet state, the scaffolds of AGG 2 and AGG3 showed slightly higher Young’s modulus values (6.52 ± 0.15 MPa and 6.26 ± 0.93 MPa, respectively) when compared to PA scaffolds (5.42 ± 0.62 MPa).

### 3.3. Cell Culture

**Figure 7:**
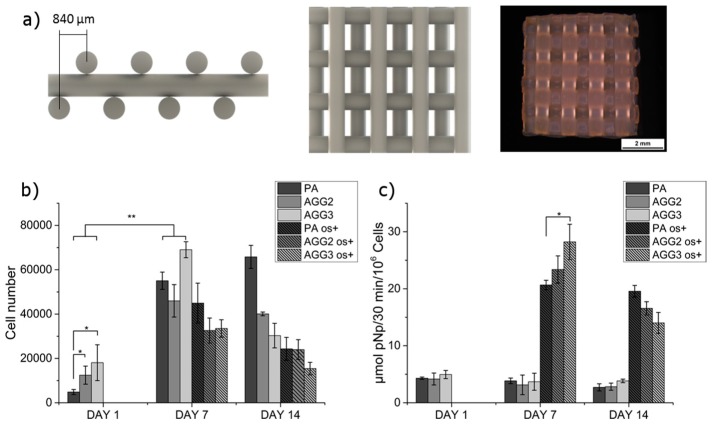
Cultivation of human mesenchymal stem cells (hMSC) on the scaffolds over a period of 14 days. (**a**) Illustration of the scaffold design with mismatch laydown pattern: CAD design (left and center) and microscopical image/top view (right); (**b**) cell number calculated from the DNA content and (**c**) specific alkaline phosphatase (ALP) activity of hMSC cultured on scaffolds when ostegenically induced (os+) and uninduced. (mean ± SD; *n* = 3; * *p* < 0.05, ** *p* < 0.005).

Number of cells attached and grown on the scaffolds were quantified by measuring the total DNA content obtained by lysis of the cells. The scaffolds used for culturing hMSC, having a mismatched architecture is shown Figure 7a. The seeding efficiency, *i.e.*, the total number of cells adhering to the scaffolds after one day of culture, was found to be higher for the composites compared to the pure alginate scaffolds (Figure 7b). After 7 days of culture, the cell number for all the scaffolds increased significantly compared to cell numbers at day 1, indicating that both pure alginate and composite scaffolds did support the cell proliferation. However, after 14 days of culture the cell number of AGG3 scaffolds had significantly reduced. Though not statistically significant, cell numbers of AGG2 had also diminished compared to cell numbers after 7 days of culture. Interestingly, an increasing trend was observed for PA scaffolds. After 1 day of culture, induction of osteogenesis was done by adding the respective supplements to the culture medium (“os+”). The stimulation of osteogenic differentiation did affect the cell proliferation: cell numbers on samples cultivated with osteogenic supplements were generally lower, however, a similar trend was visible. The specific ALP activity, an early marker for osteoblastic differentiation, was determined to evaluate if the composites support osteogenesis. Stimulated cells on AGG3 composite scaffolds showed significantly higher ALP activity in comparison to those on pure alginate scaffolds after 7 days of culture (Figure 7c). After 14 days, ALP activity of cells grown on the composites had reduced significantly in comparison to day 7. However, a constant activity was measured for cells on PA scaffolds. Also, specific ALP activity on PA scaffolds was the highest when compared to the composite after 14 days of culture.

**Figure 8:**
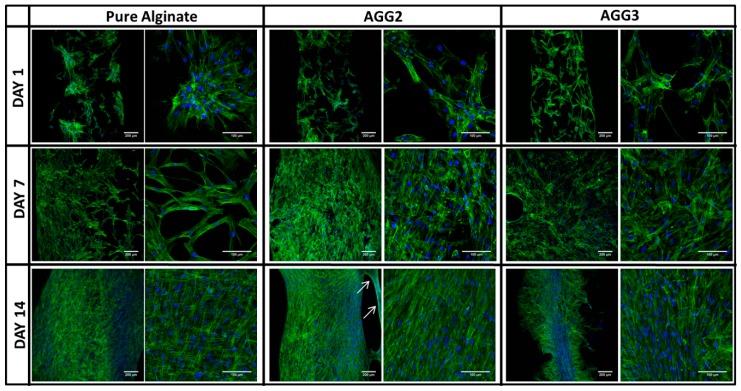
cLSM images of osteogenically uninduced hMSC cultured on scaffolds for 14 days. (blue: nucleus stained with Hoechst, green: actin cytoskeleton stained with phalloidin-AlexaFluor 488; arrows: cell sheets).

cLSM images of the cell laden scaffolds taken after 1 day showed that the cell adherence was better for the composite than for the pure alginate scaffolds (Figure 8), supporting the cell number measurements by total DNA quantification. Whereas the cells on PA scaffolds were found to be attached to the surface in clusters, a more uniform cell distribution was observed on the composite scaffolds. After 7 days of culture, the complete surface of the scaffold strands was populated by cells on the composite scaffolds. An increase in the cell numbers was evident on pure alginate scaffolds, however some un-colonized areas were also observed. Pure alginate scaffolds were completely covered by the cells after 14 days of culture. Though, AGG2 scaffold strands showed complete colonization on the strand surface, detaching cell sheets (arrows) were observed in certain areas of the scaffold at day 14. The formation of cell sheets/strands was more pronounced in the AGG3 scaffolds. It appeared that the cells were forming these cell sheets especially at the edges of the scaffolds.

## 4. Discussion

Because of its versatile gelling properties, *i.e.*, thermo-reversible gelation at near body temperature and ionotropic gelation by low cation concentrations, and the weak ability to support cell adherence, gellan gum has been investigated mainly with respect to cartilage tissue engineering applications. Low concentrated gellan gum hydrogels have also found their suitability with 3D extrusion technologies for cartilage regeneration [17,24,25], however, this is clearly limited by low shape fidelity. Print fidelity could be improved by using higher concentrations of the biopolymer which results in an increase of viscosity [14]. However, application of highly concentrated pure gellan gum pastes by AM technologies would be arduous, due to the fact that they are hard to prepare and that the thermal gelation of such a paste would be very fast leading to difficulties with the extrusion process and formation of uneven strands. Such problems can be circumvented by using composites which are usually rendered with the combination of intrinsic advantages of the individual components; however, they have to be thoroughly characterized for the intended application. Recently, we demonstrated that highly concentrated gelatin/alginate pastes can be successfully processed by 3D plotting to fabricate scaffolds of clinically relevant sizes [20]. Another strategy is to blend low concentrated alginate with a second, soluble component like methylcellulose to increase viscosity only temporarily for the plotting process. During and after alginate gelation with divalent cations methylcellulose is removed by dissolution [26]. In the present work, we prepared and applied a composite of highly concentrated alginate and low concentrated gellan gum in order to combine excellent print fidelity and favorable crosslinking ability.

The composite paste preparation was rather simple and straight forward, realized by mixing alginate powder with gellan gum solutions. The maximum concentration of gellan gum that was usable for preparing the composites was determined to be 3 wt %—higher concentrations were not applicable for composite preparation as the very fast gelling of gellan gum did not allow a homogeneous mixing with alginate, at least at room temperature. Therefore, composites with 2 and 3 wt % were prepared and evaluated. For homogeneous mixing, gellan gum solutions had to be preheated to >40 °C, however, soon after the pastes were prepared, they became more viscous and started to solidify due to the physical gelling of the gellan gum component in the composite when the temperature dropped to room temperature [3]. Though the composite pastes were partly gelled, the plotting process was not impaired; however a minimal increase in the extrusion pressure was required for the composites in comparison to pure alginate sols (Table 1). All the pastes showed a shear thinning behavior when measured till a shear rate of 100 s^−1^. This property is regarded as very important for extrusion based printers as described by Malda and co-workers [14]. A higher shear drop was observed for AGG3 and PA pastes, hence indicating that these pastes will maintain higher print fidelity [14]. Further studies involving characterizing the pastes for their other rheological properties, such as shear recovery could explain the observed difference in maintaining the shape fidelity after plotting [25]. Kesti *et al.* recently performed shear recovery studies on bioinks composed of 3% gellan gum and 2% alginate and showed that the faster shear recovery after extrusion-induced shear stress was better for the printing process. After plotting the pastes using a CAD design, the composites were crosslinked in a single step process *i.e.*, by addition of 1 M CaCl_2_ solution. In comparison to composites of alginate with gelatine [20], which requires a two-step crosslinking for stabilizing both, alginate (by addition of CaCl_2_) and gelatin (by chemical crosslinking with EDC), a single step crosslinking significantly reduced the total time for scaffold fabrication. Furthermore the cytotoxic effects of chemical (covalent) crosslinking were avoided.

A highly interconnected pore network and high total internal porosity were observed for the alginate gellan gum composites in the Nano-CT analysis of single freeze dried strands. Whereas, pure alginate strands showed a distinctive difference in terms of the pore network and total internal porosity in comparison to alginate gellan gum composites (Figure 4 and Supplementary Materials Figure S1a–c). A striking difference in the number of pores formed and pore size distribution was also observed in between the pure alginate and composite strands (Supplementary Materials, Figure S1e,f). It can be speculated that freeze drying of single *versus* double network hydrogels (*i.e.*, PA and AGG composites respectively) must be responsible for these intrinsic differences. However, in contrast to the calculated internal porosity for single strands, the total open porosity measured in the freeze-dried state of the scaffolds was found to be higher for pure alginate when compared to the composite scaffolds. High shrinkage of the PA strands during freeze drying (Figure 2d) increased the open macro pore volume and thus must have contributed to higher total porosity. Additionally, it was postulated by Haque, Kurokawa, and Gong that formation of double network hydrogels results in more stable structures in case of composites, correspondingly rendering the structures more resistant to shrinkage and swelling [27]. As a result, the macropore size of the composite scaffolds was smaller compared to PA scaffolds and thus the total porosity lower. SEM images showed that AGG3 and PA (less pronounced) scaffolds appeared to have higher shape fidelity in terms of maintaining the strand shape when compared to AGG2 scaffolds.

Swelling of the actual strand diameters in cell culture medium, HBSS (with Ca^2+^ and Mg^2+^) and PBS was important to study with respect to the suitability of the scaffolds for *in vitro* cell culture experiments and for implantation. The rationale behind quantifying the strand diameters was to characterize the variations in size of the macropores which would be an important aspect for tissue ingrowth after implantation. The swelling of pure alginate strands was higher than those of the composite strands after 24 h of incubation in medium and HBSS (with Ca^2+^ and Mg^2+^). This observation can be explained by the fact that the concentration of the divalent ions in both the solutions was very low (~5 mM of CaCl_2_) and hence the divalent metal ions from the ionically crosslinked alginate diffuse out of the matrix resulting in a weakening of the network and therefore increase water uptake and consequently the strand size [21]. In the case of the composite scaffolds, the concentration of divalent cations present in medium and HBSS (with Ca^2+^ and Mg^2+^) were sufficient for binding together the polymer chains of the gellan gum [3]. Hence, the degree of swelling was less than half of those of pure alginate strands. Though statistically not significant, the swelling ratio of composite scaffolds followed an inversely proportional trend with respect to the gellan gum concentration. However, when the composite scaffolds were incubated in PBS, more than two-fold increase in swelling was observed. The phosphate ions in PBS are known to competitively bind to divalent metal ions, forming the respective phosphate salts [28]. Hence it can be postulated that when PBS was added, a critical quantity of the Ca^2+^ ions stabilizing both alginate and gellan gum must have been released out of the matrix. As the Ca^2+^ concentration is depleted, the bridges formed in between the double helices of gellan gum polymer chains are broken inducing electrostatic repulsion between them. As a result high water uptake into the matrix must have caused the drastic increase in swelling. Potential formation of double networks in the composites further contributed to a significant difference in the swelling compared to single polymer network of PA scaffolds [27].

Mechanical strength of the scaffolds was tested by performing compressive tests on the scaffolds immediately after crosslinking, after freeze drying, and after rewetting of freeze-dried samples for 24 h in HBSS (with Ca^2+^ and Mg^2+^). For freshly crosslinked scaffolds an increase in the Young’s moduli was observed with increasing gellan gum concentration (Figure 6a). Many studies have shown that an increase in the gellan gum concentration increases the gel stiffness due to higher density of the polymer chains [6], which also explains the observed behavior of the composites when compared to pure alginate scaffolds. Additionally, higher stiffness of the gellan gum component and probable induction of double networks [29] also must have attributed to the higher mechanical strengths of the composites. Interestingly, in the dry (freeze dried) state, pure alginate scaffolds showed higher Young’s modulus values compared to the composite scaffolds. The stress strain curves of the freeze dried PA scaffolds showed a near ceramic behavior during the compressive tests. The probable reason for such a behavior could be that shrinkage of the hydrogel strands during freeze drying must have formed highly dense structures (Figure 2a). However, the composite scaffolds showed a typical stress strain curve for polymeric scaffolds (Figure 6b). Low shrinkage in the composite scaffolds (compared to PA) indicates less and inhomogeneous densification of the material leading to internal microporosity and thus attributing to lower compressive modulus. 

Cytocompatibility of highly concentrated pure alginate and the composites was studied by seeding hMSC on to the scaffolds after crosslinking and pre-incubation in cell culture medium for 48 h. The culture was carried out for 14 days with and without osteogenic supplements. After 1 day of culture, a low number of adherent cells on all type of scaffolds was observed (~10%–36% seeding efficiency). However, the composite scaffolds showed significantly higher cell numbers compared to PA scaffolds. Also, though statistically not significant, the number of adherent cells on AGG3 was higher when compared to AGG2 indicating a concentration-dependent effect (Figure 7b). Arima *et al.* showed a direct correlation between adsorption of serum proteins by carboxylic, hydroxyl, and amine groups on self-assembled monolayers (SAM’s) of alkanethiols and cell adherence [30,31]. In the present work, the amount of carboxylic and hydroxy groups should be highest in AGG3 followed by AGG2 and PA, owing to the total polymer content. Thus the amount of cell adhesive proteins adsorbed and available for cell attachment must be in the same order, resulting in highest cell attachment on AGG3 scaffolds. In the work of Rowlands *et al.* it was found that the initial hMSC attachment was higher on stiffer gels [32]. Similarly, as the stiffness of the scaffolds increased in the range PA < AGG2 < AGG3, the initial cell attachment could also be affected by this parameter. cLSM images at day 1 showed formation of cell clusters on the surface of the pure alginate scaffolds in contrast to more uniform distribution on surface of composite scaffolds, indicating that the cells preferred cell-material adherence rather than cell-cell attachment when cultured on the composites (Figure 8). After 7 days of culture, total cell number increased for both osteogenically induced and non-induced scaffolds of all types of scaffolds; cLSM images revealed a lower density of cells on the surface of PA in comparison to the composite scaffolds. However, typical spread morphology, *i.e.*, elongated cell extensions of hMSC, was observed on all the scaffolds, indicating that neither pure alginate nor the composites had impaired the cell proliferation. Interestingly, the total cell numbers measured for the composite scaffolds reduced after 14 days of culture, with higher reduction detected for AGG3 compared to AGG2 and PA. This observation is supported by cLSM images which indicated that the cell density was lower in AGG3 scaffolds at day 14. Sheet-like cell strands (Figure 8: arrows) were observed on the composite scaffolds indicating that at this time point the cells prefer cell-cell contacts and binding to matrix components, already synthesized by the cells rather than adhesion to the scaffold surface. Such a behavior was more pronounced in AGG3 scaffolds. It can be hypothesized that the amount of Ca^2+^ ions which is expected to be higher in the hydrogel matrix of the composite scaffolds and which might have led to increased Ca^2+^ release from the scaffold matrix, has an influence on cell colonization over long term culture. Accordingly, Ferris *et al.* described that the number of viable L-929 mouse fibroblasts, when cultured on gellan gum hydrogels, showed variations with respect to the concentration of Ca^2+^ used for gelation [33]. However, for elucidating these observations, further studies are needed focused on the surface stiffness, chemical composition, and cell microenvironment of the composite scaffolds.

Specific ALP activity of osteogenically induced cells was found to be higher on the composite scaffolds compared to pure alginate (Figure 7c). Furthermore, the ALP activity seemed to be increasing with increase in the gellan gum concentration after 7 days of culture. Interestingly, the ALP activity seemed to follow a completely opposite trend after 14 days of culture *i.e.*, was highest on pure alginate scaffolds. These observations indicate that osteogenic differentiation of hMSC could be accelerated on the composite scaffolds: In comparison to PA, the maximum of this early marker was achieved earlier (higher values on day 7) followed by a faster decline (lower values on day 14). The enhanced osteogenic differentiation might be associated with the formation of the agglomerating cell strands [34] and the higher local Ca^2+^ concentration [35] discussed above. The correlation of the gellan gum concentration, cell attachment and proliferation as well as ALP activity, in connection with the Ca^2+^ concentration, has to be elucidated in detail in a further study. 

Taken together, results of the cell experiments are indicative that composites of alginate and gellan gum promote early attachment and spreading of hMSC when compared to PA. Also, the composites do not negatively influence the early cell proliferation and osteogenic differentiation. Future work can be focused on improving the cell adhesion of long term cultures by optimizing the divalent cation concentrations required for crosslinking. Other divalent metal ions such as Zn^2+^ and Sr^2+^ can be used for crosslinking which might further improve the osteogenic properties of the composites, as postulated by Place and co-workers [36]. In our own studies we could demonstrate that strontium ions releasing calcium phosphate bone cements support both proliferation and osteogenic differentiation of hMSC [37,38]. Alginate as well as gellan gum and composite hydrogels of both biopolymers, ionically crosslinked with Sr^2+^ therefore could stimulate cell growth and osteogenic differentiation due to a slow release of this metal ion. Composites of bioactive glass and gellan gum have been reported to improve the mechanical strength and initial cell attachment [39]. Recently, Douglas *et al.* showed a novel method to enzymatically mineralize the gellan gum hydrogels using divalent ions (Ca^2+^, Mg^2+^ and Zn^2+^) that enhance the proliferation of osteoblast-like cells [40,41]. In context to the present work, such an approach combined with high concentration of biopolymer content and the intrinsic advantage of 3D plotting would greatly improve the applicability of gellan gum alginate composites with respect to bone tissue engineering applications, *i.e.*, cultivation of osteogenically induced cells with the aim of regenerating bone defects. Furthermore, inclusion of mineral phases in the biopolymer blends could open up the possibility of fabricating a single construct with desired gradient of mineral content and mechanical strengths, along with the ability to support both osteoblasts and chondrocytes required to repair osteochondral defects.

## 5. Conclusion

Composites of alginate and gellan gum have shown to be better suitable for fabrication of 3D plotted scaffolds. Addition of gellan gum had shown to improve shape fidelity and decrease the swelling in cell culture medium. Also an increase in the mechanical strength (in wet state) was observed for composite scaffolds compared to pure alginate scaffolds. Initial hMSC attachment on the composites was found to be favorable and did not negatively influence osteogenic differentiation. However, the cell numbers had reduced after two weeks of culture. Addition of or coating with RGD peptides or utilizing other divalent cations (such as Sr^2+^ or Zn^2+^) for crosslinking might further improve cell behavior on the alginate-gellan gum composites in long term cell cultures.

## Figures and Tables

**Table 1:** Average viscosity at constant shear rate of 10 s^−1^ and the extrusion pressure of the pastes.

Material	Average viscosity (Pa s) (at constant shear rate: 10 s^−1^)	Extrusion pressure (kPa)
PA	530.2 ± 6.3	480
AGG2	738.6 ± 10.4	520
AGG3	843.2 ± 9.9	530

**Table 2:** Total porosity of freeze dried scaffolds measured by pycnometer and micro-computed tomography (µ-CT) data analysis. Porosity measured by pycnometer showed a statistically significant (** *p* < 0.005) difference between values of PA compared to AGG2 and AGG3.

Scaffold type	Porosity measured by pycnometer (*n* = 5) (%)	Porosity measured by µCT image analysis (*n* = 4) (%)
PA	79.7 ± 1.2	66.9 ± 3.4
AGG2	69.5 ± 1.2 **	60.8 ± 2.8
AGG3	69.3 ± 1.0 **	62.3 ± 2.3

## Supplementary Materials

Supplementary materials can be found at www.mdpi.com/2073-4360/8/5/170/s1.
