# Supplementary material for: Highly Concentrated Alginate-Gellan Gum Composites for 3D Plotting of Complex Tissue Engineering Scaffolds
Source: Polymers (Basel). 2016 Apr 26;8(5):170. doi: 10.3390/polym8050170 (PMC6432352; doi:10.3390/polym8050170)
Supplement: Supplementary file 1 [file polymers-08-00170-s001.pdf]

Polymers **2016**, *8*, 170; doi:10.3390/polym8050170

Supplementary Materials: Highly Concentrated Alginate/Gellan Gum Composites for 3D Plotting of Complex Tissue Engineering Scaffolds

Ashwini Rahul Akkineni, Tilman Ahlfeld, Alexander Funk, Anja Waske, Anja Lode and Michael Gelinsky

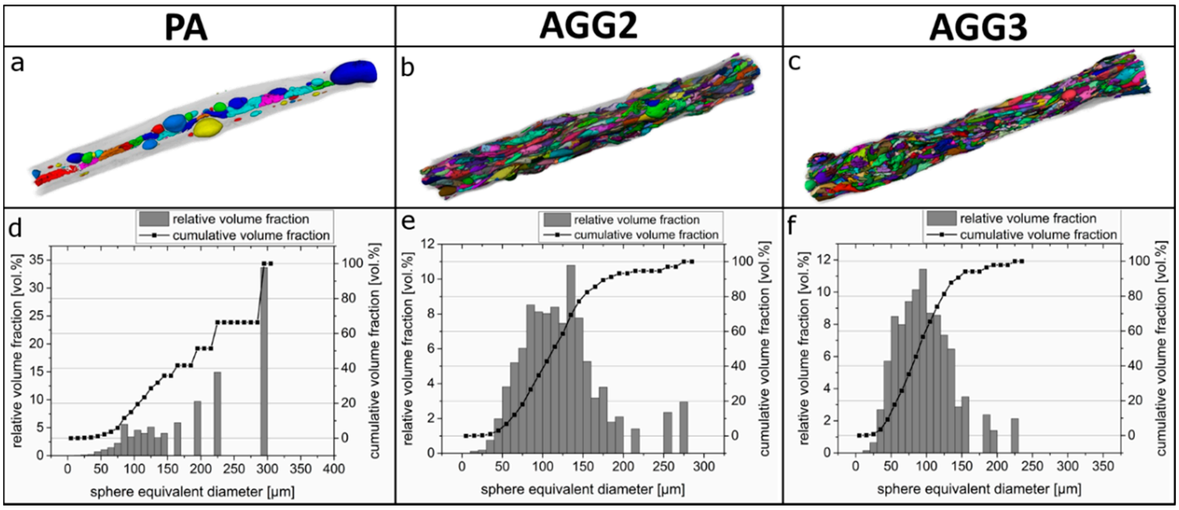

Figure S1. Pores in the strands visualized by reconstructed Nano-CT scans of single freeze dried strands (**a**) PA; (**b**) AGG2; (**c**) AGG3 (colors indicate single separated pores). The length of the strands was 4 mm. Pore size distribution of (**d**) PA; (**e**) AGG2; (**f**) AGG3 strands.

© 2016 by the authors; licensee MDPI, Basel, Switzerland. This article is an open access article distributed under the terms and conditions of the Creative Commons Attribution (CC-BY) license (http://creativecommons.org/licenses/by/4.0/).
